# CLPB Deficiency, a Mitochondrial Chaperonopathy With Neutropenia and Neurological Presentation

**DOI:** 10.1002/jimd.70025

**Published:** 2025-04-07

**Authors:** D. Mróz, J. Jagłowska, R. A. Wevers, S. Ziętkiewicz

**Affiliations:** ^1^ Intercollegiate Faculty of Biotechnology University of Gdansk Gdansk Poland; ^2^ Department of Pediatrics, Hematology and Oncology Medical University of Gdansk Gdansk Poland; ^3^ Department of Human Genetics Radboud University Medical Center Nijmegen the Netherlands

**Keywords:** 3‐methylglutaconic aciduria, CLPB, congenital neutropenia, disaggregase, mitochondrial chaperonopathy

## 1 Introduction

Human CLPB protein, a mitochondrial disaggregase, gained recognition in 2015, when four independent studies identified pathogenic variants in *CLPB* as the underlying cause of a novel autosomal recessive multiorgan disorder presenting distinct laboratory findings, namely neutropenia and 3‐methylglutaconic aciduria (ORPHA:445038; MIM #616271) [1, 2, 3, 4]. Biallelic *CLPB* deficiency is a neurodevelopmental disorder with neutropenia and cataracts. More recently, certain monoallelic disease‐causing germline variants were further identified as the cause of autosomal dominant severe congenital neutropenia (CLPB‐SCN; ORPHA:486; MIM #619813) [5, 6, 7]. Newest in the field is the discovery that also a few monoallelic variants cause the same phenotype as in biallelic *CLPB* defect with the exception of cataracts [5] (MIM #619835).

The *CLPB* gene is located on chromosome 11 (11q13.4) and consists of 19 exons. Four isoforms have been reported at the mRNA level, with two major isoforms reported at the protein level. The protein was named CLPB due to the surprising similarity between its AAA+ domain and the second AAA+ domain of bacterial ClpB (itself misnamed ‘Caseinolytic protease type B’ for similarity to ClpA protease, before it was characterised as a disaggregase without proteolytic activity) and its yeast orthologues Hsp104 and Hsp78 [4].

Though the study of the human protein gained momentum only recently, a mammalian CLPB orthologue was first described by Périer et al. [8], where the expression of cDNA of the mouse CLPB homologue rescued the phenotype of the potassium‐dependent yeast Trk‐ growth defect. The protein was thus named Skd3 (suppressor of K+ transport growth defect 3).

## 2 CLPB Protein Structure and Function

**FIGURE 1:**
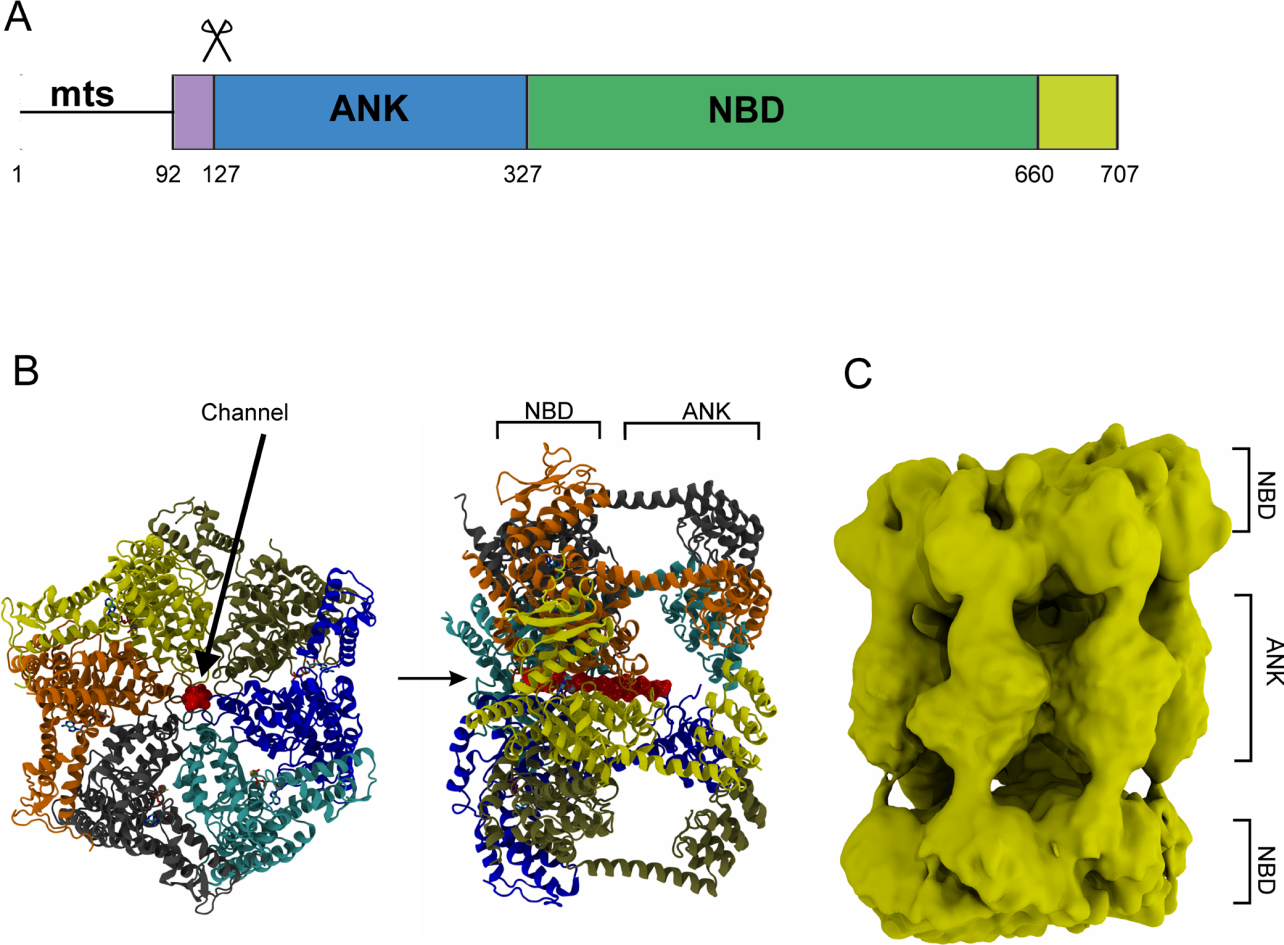
Structural features of CLPB. (A) Domain composition of CLPB: mts: mitochondrial targeting sequence, ANK, region of four ankyrin repeats, NBD, nucleotide binding domain. Scissors denote PARL cleavage site removing autoinhibitory peptide. (B) CLPB protein structure. Left, CLPB hexamer (PDBID:7TTS [10]) seen along (left) and perpendicular (right) relative to central channel axis. ANK, NBD—positions of ankyrin repeats and nucleotide binding domain, respectively. Central channel occupied by a substrate chain (red) marked by arrow. (C) Cryo‐EM structure of CLPB dodecamer, consisting of two hexameric rings joined by ankyrin fragments (Electron Microscopy DataBank EMD‐26728 [11]).

CLPB is a mitochondrial AAA+ ( *A* TPases *A* ssociated with diverse cellular *A* ctivities) ATPase containing an HCLR‐type ( *H* slU, *C* lp‐D2, *L* on and *R* uvB) ATPase domain characteristic for Hsp100 disaggregases. Similarly to those disaggregases, human CLPB in vitro solubilises aggregated luciferase, a model substrate and refolds it into enzymatically active form, implying a mitochondrial chaperone function [9]. The ATPase domain is, however, preceded by a series of ankyrin repeats (Figure 1) which is an unprecedented feature in this group.

AAA+ proteins are involved in a wide variety of cellular processes, including proteolysis, DNA replication and repair, membrane fusion and protein unfolding and disaggregation. The proteins from this group usually form homohexameric rings and utilise the energy from ATP hydrolysis to thread their substrates through the central pore formed by the subunits. The characteristic feature of the ATPase domain of AAA+ proteins is the presence of Walker A (P‐loop) and Walker B motifs, responsible for ATP binding and hydrolysis. Additional motifs highly conserved in AAA+ proteins are Sensor 1, Sensor 2 and the arginine finger [12]. Apart from these, CLPB has a protruding loop (residues L507‐I534), which is also conserved among CLPB homologues. Its deletion results in increased ATPase and disaggregase activity [10]. It is postulated that the loop might have a regulatory function and is a likely candidate for an interaction site.

What distinguishes human CLPB from its non‐eukaryotic orthologs, yeast Hsp104 and bacterial ClpB, is the presence of an ankyrin domain comprising a series of ankyrin repeats. The ankyrin region of CLPB consists of two pairs of ankyrin repeats separated by a linker, whose length equals that of two ankyrin motifs. The linker sequence shows some limited homology to the consensus sequence of ankyrin repeats. Ankyrin repeats 1–3 adopt a canonical structure, whereas the structure of ankyrin repeat 4 is degenerated. It is also directly connected to the ATPase domain of the protein without any linker structure separating the two domains [13]. Isoforms 1 and 2 of CLPB differ in the length of the linker between repeats 2 and 3.

Our group was the first to demonstrate the ATPase activity of the canonical CLPB isoform 1 [4]. We have also shown that the protein can efficiently disaggregate firefly luciferase aggregates, but not aggregated GFP. In contrast to bacterial ClpB and yeast Hsp104, human CLPB does not require cooperation of Hsp70/40 for its disaggregase activity; but, on the contrary, competes with them for aggregate binding [9].

Recent studies showed that a shorter CLPB form resulting from cleavage by PARL protease is the physiological form of the protein and has a higher disaggregase activity than the precursor form [14]. Additionally, PARL CLPB exhibits some disaggregation activity towards α‐synuclein amyloid fibres, whose aggregation is directly implicated in Parkinson's disease and other neurological disorders [13, 14]. The ATPase activity of CLPB isoform 2 is half that of isoform 1, whereas the disaggregase activity is higher [13, 15]. This difference in disaggregation activity is especially visible for α‐synuclein fibres [13]. Interestingly, CLPB prevents fibril formation by amyloid β peptide in vitro [11]; some protective effect could be observed with the isolated ankyrin domain. The exact role of CLPB and its isoforms in the amyloid fibre assembly prevention remains to be elucidated.

The expected oligomeric structure of CLPB was a hexamer, as it is the most common form for AAA+ proteins, bacterial ClpB and yeast Hsp104 included. However, even in the absence of precise structural data, our early studies pointed to a larger structure of human CLPB, probably dodecameric. In Mróz et al. [9], we presented data from size exclusion chromatography, atomic force microscopy and negative‐stain electron microscopy suggesting the formation of such higher‐order oligomers. The first precise data on the oligomeric state of CLPB was obtained by Spaulding et al. [16] They established that at low protein concentrations the addition of a nucleotide shifts the estimated molecular weight of the observed protein species around 12 times, as does increasing the protein concentration. The estimated weights of the two species are consistent with a monomeric and a dodecameric form.

Cupo et al. [10] presented several cryo‐EM images (Figure 1B) further elucidating the structure of CLPB. The cryo‐EM data was obtained in the presence of a model substrate, FITC‐casein. The captured species were characterised by a hexameric ring with a central pore as observed in the top view, and two or three stacked layers in the side view. The two‐tiered, more prevalent species was assumed to be a hexamer, with a well‐resolved ring corresponding to the nucleotide‐binding domain, and a second ring with poorer resolution, containing separated and flexible globular entities consistent with the presence of ankyrin repeats.

The suggested explanation for the three‐tiered structure is a dimer of hexamers organised in a head‐to‐head manner, with the interactions between the hexamers provided by the ankyrin repeats. With the prevalence of dodecamers observed in size exclusion chromatography and independently reported by several studies, and the hexameric form dominating in the cryo‐EM images, CLPB is purported to exist in a dynamic equilibrium between the two forms.

Hexamers of CLPB nucleotide binding domains adopt a quaternary structure typical for AAA+ proteins [12] while the ankyrin domain is crucial for dodecamerisation, as it mediates contact between two hexamers (Figure 1C). In line with this model, it is presumed that the substrate is handed over through the central pore of the AAA+ machine.

While the structure and substrate processing by the CLPB hexamer are typical for AAA+ proteins, the physiological function of the dodecamer remains unresolved. Gupta et al. [11] prepared artificial CLPB constructs with impaired dodecamer formation, which demonstrated diminished refolding, but not solubilisation of aggregated luciferase. Consequently, they proposed a two‐step model of the refolding activity of CLPB, whereby disaggregation of a polypeptide is followed by its refolding within a protective cage formed by the dodecamer.

## 3 CLPB Protein Biology

CLPB is a mitochondrial protein with an N‐terminal mitochondrial targeting sequence (MTS). It is located exclusively in the mitochondrial intermembrane space (IMS) [6, 17, 18, 19, 20, 21, 22, 23], and has been proposed as a marker protein for this subcompartment [19]. In the IMS, CLPB is closely associated with the inner mitochondrial membrane (IMM) [6, 20, 22, 23]. Its depletion results in decreased solubility of a variety of IMS and IMM proteins, mainly involved in apoptosis, mitochondrial import, oxidative phosphorylation and mitochondrial calcium homeostasis [14, 23], proving the physiological significance of the disaggregase function of CLPB. Additionally, CLPB has been shown to physically interact with a variety of IMS and IMM proteins playing crucial roles in mitochondrial function.

CLPB is a substrate of the IMM rhomboid protease PARL, which cleaves it at position 127 [24], removing a hydrophobic peptide and decreasing the disaggregase activity of the protein, likely targeting CLPB to the IMS. Additionally, CLPB co‐precipitates with both wild‐type and protease‐deficient PARL [24, 25], which suggests interactions beyond a protease‐substrate relationship. Furthermore, PARL forms a multiprotein SPY complex with YME1L1 (i‐AAA+ protease) and STOML2 [23, 25]. CLPB associates with all members of the SPY complex and influences their solubility, stability and activity [23].

The most important CLPB interactor is the multifunctional protein HAX1, inter alia a major regulator of myeloid homeostasis. Similarly, CLPB is the most prominent mitochondrial partner of HAX1 [6, 26]. Biallelic disease‐causing mutations in *HAX1* underlie Kostmann syndrome [27, 28] (ORPHA:99749; MIM #610738), manifesting as severe congenital neutropenia with occasional neurological symptoms. Due to the partial overlap with the symptoms of *CLPB* mutations, a link between the two proteins has been long postulated [4]. It has been shown that CLPB ablation drastically reduces HAX1 solubility [6, 13, 14, 23], suggesting their relationship relies on the disaggregase activity of CLPB. Direct interaction between the proteins has since been demonstrated in vitro and in vivo [6, 23, 26, 29, 30]. HAX1 is the only protein whose precise site of interaction with CLPB has been identified. The interaction site has been mapped to HAX1 residues 126–137. Pathogenic variants affecting this region are known to correlate with the presence of neurological symptoms in addition to severe congenital neutropenia. For example, the *HAX1* c.389T>G (L130R) variant, clinically resulting in neutropenia with neurological involvement, abolishes the interaction between HAX1 and CLPB. As neurological manifestations in Kostmann syndrome result from disrupted HAX1‐CLPB interaction, and deletion of either protein leads to a similar cellular phenotype, HAX1 has been proposed as a downstream effector of CLPB [6]. Additionally, CLPB and HAX1 share interaction partners, including PARL [31, 32] and Prohibitin2 [33] (PHB2), which strongly suggests their cooperation in various processes in the IMS.

Another shared interactor of HAX1 and CLPB is the protease HTRA2. HAX1 delivers HTRA2 to PARL for its proteolytic activation and acts as both its substrate and its allosteric activator [31, 32, 34]. An interaction between HTRA2 and CLPB itself has been demonstrated with co‐immunoprecipitation, and the solubility of HTRA2 significantly decreases in the absence of CLPB [14, 23]. This relationship might be physiologically relevant, as biallelic pathogenic variants in the *HTRA2* gene underlie 3‐methylglutaconic aciduria type VIII, sharing symptoms with *CLPB* deficiency [35] (ORPHA:505208; MIM #617248;) while heterozygous variants have been postulated as a susceptibility factor in a subset of early‐onset Parkinson disease (MIM #610297).

CLPB has also been demonstrated to interact directly with the OPA1 mitochondrial dynamin‐like GTPase (formerly: optic atrophy protein 1), a key component of mitochondrial cristae [26] whose malfunction is responsible for the development of a progressive neuro‐ophthalmological disease, Kjer optic atrophy (ORPHA:98673; MIM#165500). Mitochondrial cristae morphology is maintained by membrane‐bound oligomeric structures composed of the membrane‐anchored long forms (L‐OPA1) and the soluble short forms (S‐OPA1), resulting from proteolytic cleavage of L‐OPA1 [36, 37, 38, 39]. CLPB ablation results in an overall increase in the level of OPA1 and accumulation of S‐OPA1 [23, 26]. Additionally, the similar disruption in mitochondrial morphology upon deletion of either protein suggests their cooperation in cristae maintenance [26].

Another example of CLPB protein partners involved in maintaining the proper mitochondrial architecture is prohibitins. Prohibitins PHB1 and PHB2 form multimeric ring‐shaped structures in the IMM. Their loss results in abnormal cristae morphology, inhibition of mitochondrial fusion, fragmentation of the mitochondrial network and increased susceptibility to apoptotic stimuli [40, 41]. Prohibitins are putative scaffolds and regulators of various processes. They participate in membrane organisation and the creation of functional microdomains in the IMM, regulating interactions within the membrane [42]. They are often found in supercomplexes with IMM‐anchored or associated proteins, influencing respiratory complex formation and stability, and OPA1 processing [38, 43, 44, 45, 46, 47, 48]. Apart from that, prohibitins participate in signal transduction in the mitochondria [49], including the RIG‐I antiviral pathway, where they cooperate with CLPB [20].

The interactors of CLPB are summarised in Table S3.

The data for CLPB disruption on the cell proteome is given in a Table S2.

## 4 Genetic Disorders Resulting From *CLPB* Gene Defect

CLPB (MIM *6162540) deficiency is a rare mitochondrial disorder characterised by severe neutropenia and progressive neurologic manifestations of variable severity, from the mildest cases reaching adulthood and capable of independent living to the most severe ending in death in the neonatal period [1, 2, 3, 4, 50, 51, 52, 53, 54, 55]. Its laboratory hallmark is the presence of 3‐methylglutaconic aciduria observed in the majority of patients. Consequently, CLPB deficiency was assigned to the group of secondary 3‐methylglutaconic acidurias (MGAs), a class of inherited metabolic disorders with an excessive urinary level of 3‐methylglutaconic acid and neurodevelopmental phenotypes as their discriminatory feature, and was given the name 3‐methylglutaconic aciduria type VII (MGA7). The mechanisms leading to elevated 3‐MGAs in CLPB deficiency remain, however, unknown. Conversely, primary 3‐methylglutaconuria (MGA1; MIM*250950) is an organic aciduria due to a leucine metabolism defect, as a consequence of biallelic pathogenic variants in the *AUH* gene encoding 3‐methylglutaconyl‐CoA hydratase.

The phenotype associated with *CLPB* deficiency (*aka*. MGA7) initially reported as occurring due to a biallelic gene defect was later also identified in a series of patients with *de novo* heterozygous missense pathogenic variants severely affecting the disaggregase and to a lesser degree the ATPase activity of CLPB in a dominant‐negative manner [5]. Accordingly, CLPB deficiency was further subdivided with respect to the inheritance pattern into autosomal dominant (MGA 7A; MIM#619835) and autosomal recessive (MGA 7B; MIM#616271). It should be underlined that variants causative for the dominant disease have not been reported in families with recessive disease nor in populational databases, and all occurred as a de novo events [5].

Although neurological manifestations are typical for all MGAs, the distinctive features of *CLPB* deficiency are bilateral congenital or infantile cataracts (present in MGA 7B) and neutropenia, which can range from severe to mild. Recent studies add premature ovarian insufficiency (POI) and male infertility (oligospermia/azoospermia) as a long‐term sequel [53]. Conversely, optic nerve atrophy and cardiomyopathy often observed in other forms of secondary MGAs have not been reported so far in CLPB patients.

In individuals with CLPB deficiency, the most common neurological findings are developmental delay and/or regression, intellectual deficits and neonatal hypotonia, which could later lead to spasticity, seizures and a progressive movement disorder (ataxia, dystonia and/or dyskinesia). The most severe cases additionally present a lack of voluntary movements, with ventilator dependency and a hyperekplexia (hyperexcitability to tactile stimuli) as an effect of progressive cerebellar atrophy, sometimes accompanied by cerebral atrophy leading to early death [3, 5, 51].

White matter lesions associated with CLPB deficiency are attributable to mitochondrial dysfunction, which in turn contributes to oxidative stress and oligodendrocyte apoptosis. This is a probable pathomechanism for severe frontal cystic leukoencephalopathy in patients with biallelic stop‐gain mutations in CLPB (c.1159C>T; R387*) [55].

It should be noted that the majority of patients with causative monoallelic *CLPB* variants reported so far present with isolated neutropenia without other symptoms characteristic of *CLPB* deficiency [5, 6, 7]. These patients were originally classified as having a novel type of (isolated) severe congenital neutropenia (SCN type 9 *aka*. CLPB‐SCN; MIM #619813; ORPHA:486). It remains to be established whether the distinction between dominant CLPB deficiency (MGA 7A) and CLPB‐SCN is scientifically valid or if these entities represent, in fact, one disorder. 3‐MGA‐uria, distinctive for the aforesaid *CLPB* deficiency, was present in a minority of monoallelic *CLPB* individuals—only a few among those described by Wortmann et al. [5] and referred to in their study as a dominant *CLPB* deficiency. The distinction based on the lack of 3‐MGA‐uria in isolated *CLPB*‐related neutropenia needs to be further studied, as this parameter was not assessed in 9 out of 20 published cases, since it had not been linked with neutropenia before. The issue of 3‐MGA presence in SCN is particularly puzzling in the case of mutations of the arginine finger R561. Mutations of this residue have been reported in nine unrelated patients, the largest number for any CLPB amino acid. Moreover, three different pathogenic variants have been reported for this residue—R561G, R561Q and R561W. Patients harbouring the R561W variant have elevated urinary levels of 3‐MGA, whereas patients with R561G and R561Q variants do not [5, 7].

The monoallelic SCN‐related *CLPB* pathogenic variants are located near the C‐terminal ATP‐binding domain and are predicted to interact with the ATP‐binding pocket [7]. Their lentiviral expression, but not that of the recessive R408G variant, resulted in impaired mitochondrial respiration in MOLM‐13 cells. This effect is distinct from the one observed in CLPB‐related 3‐MGA‐uria, where the gene defect had no effect on respiration efficiency [4].

So far, 39 patients with biallelic pathogenic variants in *CLPB* (MGA 7B) have been described [1, 2, 3, 4, 50, 51, 52, 53, 54, 55], whereas for the dominant variants (MGA 7A and CLPB‐SCN) 20 cases have been reported [5, 6, 7]. The phenotypic spectrum of the disease is presented in Table 1.

**TABLE 1:** Phenotypic continuum of CLPB deficiency.

Features	*CLPB*‐biallelic (3‐methylglutaconic aciduria, type VIIB, autosomal recessive; OMIM# 616271)	*CLPB*‐monoallelic‐neurodevelopmental disorder (NDD) phenotype (3‐methylglutaconic aciduria, type VIIA, autosomal dominant; OMIM # 619835)	*CLPB*‐monoallelic‐isolated neutropenia phenotype (neutropenia, severe congenital, 9 (SCN9), autosomal dominant; OMIM # 619813)
Neutropenia	31/37 (83%)	6/7 (86%)	13/13 (100%)
Cataract	15/39 (38%)	—	1/13 (8%)
Hypotrophy	12/39 (30%)	—	1/13 (8%)
Microcephaly	15/39 (38%)	5/7 (71%)	—
Brain atrophy (MRI, autopsy)	16/32 (50%)	6/7 (86%)	—
Facial dysmorphisms	6/39 (15%)	1/7 (14%)	—
Respiratory failure	7/39 (17%)	—	—
Death	20/39 (51%)	1/7 (14%)	1/13 (8%)
*Biochemical hallmark*			
Urinary 3‐MGA	34/35 (97%)	6/6 (100%)	0/5 (0%)
*Neurological symptoms*			
Seizures	21/37 (56%)	5/7 (71%)	2/13 (20%)
Altered muscle tone (hypotonia or spasticity)	18/39 (46%)	6/7 (86%)	—
Movement disorder (ataxia, dystonia, hyperkinesia, hyperekplexia)	20/37 (54%)	—	—
DD/ID	31/39 (79%)	7/7 (100%) (1‐mild, 4‐moderate, 2‐severe)	1/13 (10%)
No of patients	39	7	13

**FIGURE 2:**
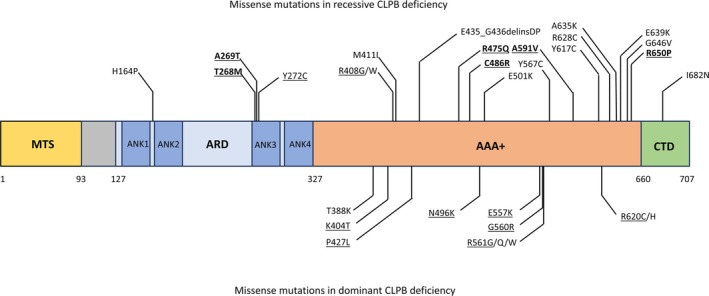
Map of CLPB pathogenic missense variants. Map of the missense pathogenic variants occurring in recessive (upper part) and dominant (lower part) *CLPB* deficiency. Variants reported in homozygosity are in bold, and protein variants analysed biochemically are underlined.

The mutations underlying the recessive CLPB deficiency are found in all regions of the protein (Figure 2). Most of the encountered pathogenic variants are missense; however, nonsense and frameshift mutations are observed as well, as are splicing defects [53, 54]. Most of the described cases are compound heterozygotes; however, several variants have been observed in homozygosity.

The analysed mutations related to the autosomal dominant disorder are as follows: K404T, P427L, N496K, E557K, G560R, R561G, R561Q, R561W and R620C. All these variants affect the NBD domain of CLPB. Apart from K404T, which is located around the intersubunit interface, in the vicinity of the recessively transmitted R408G, the mutations occur around functionally relevant regions of the ATPase. P427L lies in the direct neighbourhood of the primary pore loop, N496K mutates the sensor‐1 motif and R620C affects the sensor‐2 motif. E557K, G560G, R561G and R561Q are located in the conserved stretch including the arginine finger, with R561G and R561Q affecting the arginine finger itself. A comprehensive list of variants associated with all three disorders resulting from CLPB malfunction is presented in Table S1 and Figure 2.

The reasons determining whether a specific variant results in the recessive or dominant disease are currently unknown. Interestingly, some of the dominant variants occur in the close vicinity of the recessive ones, as is the case with K404T (dominant)—R408G/W (recessive), N496K (dominant)—E501K and Y617C (recessive)—R620C/H (dominant)—R628C (recessive) (Figure 2). A perplexing issue is why the SCN‐related variants, with such a devastating influence on the ATPase and disaggregase activity on the wild‐type protein in vitro, result in a relatively mild phenotype in patients. Then again, the K404T variant associated with the MGA 7A phenotype (dominant CLPB deficiency) did not manifest neutropenia [5].

## 5 Characteristics of Pathogenic *CLPB* Variants Present in Affected Individuals

Several disease‐causing CLPB missense variants, both dominant and recessive, have been characterised at the biochemical level [4, 5, 6, 7, 10, 11, 13, 14].

The oligomeric status has been described for the dominant variants K404T, P427L and G560R, which were assessed as a 1:1 mix with the wild‐type protein. None of the mutations abolish hexamer or dodecamer formation; nevertheless, some variation is observed in the equilibrium between the two forms. ATPase and disaggregase assays were performed for all variants. For the recessive mutations, the ATPase activity might be lower, similar or even higher than that for the wild‐type protein. In the case of dominant mutations, however, the ATPase activity is always lower, with the best performing p.P427L exhibiting activity at 60% of the wild type. In contrast to the ATPase activity, the disaggregase performance of both recessive and dominant variants is affected. For the recessive variants, the degree of disaggregase impairment correlated with disease severity [14]. The dominant mutations, K404T, P427L, N496K, E557K, G560R, R561G and R620C, also decreased the disaggregase activity of the wild‐type subunits in mixed oligomers. For all analysed variants, the observed disaggregase activity was lower than 50% of the activity of the wild type protein, which demonstrates that the dominant phenotype of the disorder is brought about by the inhibitory effect of mutant subunits on the wild type ones in mixed oligomers.

Fan et al. [6] studied the influence of the recessive T268M, Y272C, R408G and dominant R561Q variants on the interaction with HAX1. Out of these, only Y272C did not bind HAX1. C272, resulting from the mutation, might form an abnormal disulphide bridge with C267 [13] thus impacting the folding of the ankyrin domain, identified as the HAX1 binding site.

## 6 CLPB in Other Clinical Entities

Apart from MGA7 and SCN9, which are a direct consequence of CLPB malfunction, the protein has been implicated in a variety of additional pathologies.

CLPB is implicated in the development and progression of acute myeloid leukaemia (AML). *CLPB* is upregulated in this cancer type in general, and especially in cells displaying resistance to venetoclax [26]. The role of CLPB in other cancers is less known; however, recent studies point to its involvement in the pathogenesis of solid tumours too. A case in point is castration‐resistant prostate cancer (CRPC); here, expression of the *CLPB* gene is negatively correlated with progression‐free survival [56].

Another area of disease‐related CLPB activity is the process of immune response to infection with RNA viruses, which relies on the interaction between CLPB and prohibitin PHB2. During the infection, mitochondrial antiviral signalling protein (MAVS) oligomerises and forms a multiprotein complex involving CLPB and PHB2, which activates the RIG‐I (retinoic acid‐inducible gene I) signalling pathway, a mitochondrially mediated antiviral innate immune response. Interestingly, the interaction between CLPB and MAVS occurs only in the presence of PHB2 [20].

## 7 Effects of CLPB in the Oxidative Phosphorylation System

The role of CLPB in cellular respiration is far from clear. Given that it safeguards the solubility of subunits and assembly factors of respiratory supercomplexes, CLPB likely participates in the maintenance of the oxidative phosphorylation system. Consequently, its defect should impair cellular respiration, as does the malfunction of many mitochondrial proteins.

However, the data obtained from patient cells paints a different picture. We have previously [4] reported no decrease in the OXPHOS activity in patient fibroblasts. Saunders et al. [3] analysed the activity of the respiratory complexes in the liver of a patient harbouring two nonsense alleles. The observed activity was normal except for Complex III, whose activity was markedly decreased. Accordingly, Tucker et al. [53] observed no decrease in the abundance of OXPHOS components in the lymphoblasts derived from a patient with a biallelic CLPB defect.

On the other hand, proteomic analyses of different CLPB‐null cell lines revealed differences in the abundance, synthesis, persistence and solubility of various components and assembly factors of respiratory complexes [6, 14, 23].

Discrepancies between cellular models and patient cells can also be observed regarding the activity of respiratory complexes. Respiration efficiency was significantly decreased in CLPB‐null HEK293T and MOLM‐13 cells, as well as in native MOLM‐13 cells supplemented with exogenously expressed SCN‐related *CLPB* variants.

## 8 Future Perspectives

Though the body of CLPB research is continuously expanding and its further aspects are receiving attention, the study of this protein is still in its initial stages. Consequently, there are still considerable gaps regarding the precise mode of action of CLPB in the cell, as well as the mechanism of its malfunction in disease.

To start with, it should be yet again underlined that the surprising feature of CLPB is its dodecameric structure. Though not entirely uncommon for an AAA+ protein, a dodecameric member of the Hsp100 family had not been encountered before. While the dodecamer seems to be the dominant species, the hexamer and dodecamer exist in an equilibrium in vitro. What remains to be established is the physiological significance of the two forms, including whether they play different roles in the disaggregation process, or under which conditions the switch between the two species occurs.

The second crucial issue of CLPB‐related research is identifying its *bona fide* substrates. Good candidates for substrates can be found among the proteins with reduced solubility in the absence of CLPB [14, 23]. Some of these proteins are direct clients of CLPB, while the decreased solubility of others could result from disrupted proteostasis in the IMS and IMM. A case in point is that CLPB ablation drives the reduced solubility of the proteolytic SPY complex [23]. Decreased activity of a major proteolytic hub could be crucial for the total solubility in the IMS, generating more insoluble proteins than would result from the absence of a disaggregase alone.

Thirdly, no mechanism of regulation has been discovered for CLPB so far. Yeast Hsp104 is regulated by Hsp70 binding to its M‐domain and overcoming the inhibition. A constitutively active Hsp104 variant D484K is hyperactive and toxic to the cell, as it is able to unfold native proteins [57]. As the activity of PARL‐processed CLPB reminds that of Hsp104, especially for isoform 2, the uncontrolled action of CLPB could potentially wreak havoc in the IMS environment by uncontrolled unfolding.

The role of CLPB in the cell is its involvement in cellular respiration remains yet to be fully explained. It seems that neither the ATPase nor the disaggregase activity of CLPB is necessary for mitochondrial respiration. A case in point is the A591V mutation, which abolishes both the ATPase and disaggregase activity of CLPB but, on the other hand, does not impair mitochondrial respiration in the fibroblasts of a homozygous patient [4]. Even more puzzling is the activity of the OXPHOS system in the liver of a patient with two nonsense alleles, which was reduced solely for Complex III [3]. The activity of the oxidative phosphorylation system is the major difference between patient‐derived cells and cellular models of CLPB defect. Neither CLPB knockout nor overexpression of the dominant negative pathogenic CLPB variants accurately reflect the respiratory phenotype of patient cells. The discrepancy is especially glaring in the case of the CLPB‐related SCN patients, who exhibit a relatively mild phenotype, frequently consisting of isolated neutropenia that is often manageable with G‐CSF. In contrast, MOLM‐13 cells overexpressing selected SCN‐related CLPB variants in addition to the native wild‐type protein, which were studied as a model of the disease, demonstrated severely impaired respiration [7].

Subunits and assembly factors of various respiratory complexes are among the proteins whose solubility is significantly decreased in the absence of CLPB. Yet still, further studies are needed to demonstrate whether the stabilising effect of CLPB is a direct result of its disaggregase activity.

Lastly, the analysis of CLPB activity is complicated by the existence of at least two isoforms likely to play a significant role in the cell. The accumulating evidence of the importance of CLPB isoform 2 calls for investigation of the distribution of isoforms 1 and 2 in different tissues and cell lines, which could contribute to understanding which isoform is affected in CLPB defects. The presence and role of isoform 3 still need to be demonstrated. So far it has only been reported on the mRNA, but not at the protein level. It might be the case that it is tissue or development stage‐specific and that is why it has still not been detected. The elucidation of spatial and temporal expression of CLPB, its tissue and isoform expression profile, appears then as one of the key questions. Especially, the central nervous system expression is intriguing considering the phenotypic spectrum.

The physiological role of microbial disaggregases like ClpB of *Escherichia coli* , after which CLPB was named, is a reactivation of proteins aggregated during heat shock, ensuring the viability of this condition. While this is hardly the case for human CLPB, its disaggregation activity needed in mitochondrial proteostasis may be responsible for the variable clinical outcome and obscure the genotype–phenotype correlation. Clinical outcome may therefore heavily depend on the stability of other proteins in an individual's genetic constellation as well as on the nature of the defect in question itself. Diseases caused by CLPB dysfunction may be considered chaperonopathies.

At present, due to the limited knowledge of the molecular basis of CLPB‐related diseases, the only available treatment is symptomatic. Further studies elucidating CLPB biochemistry, its substrates, and regulators will not only pinpoint its role in cellular physiology but possibly lead to the discovery of therapeutic targets and, eventually, precision medicine for the patients.

### 8.1 Note

In this work, CLPB residues are numbered according to UniProt Q9H078 and NP_110440.1. Please note that the recent update of MANE Select proposed changes that result in altered nucleotide and predicted protein position numbers (https://www.ncbi.nlm.nih.gov/refseq/MANE/).

## Ethics Statement

This article does not contain any studies with human or animal subjects performed by any of the authors.

## Conflicts of Interest

Ron A. Wevers declares that he is Chair of the Scientific Advisory Board of Metakids. Dagmara Mróz, Joanna Jagłowska, Szymon Ziętkiewicz declare that they have no conflicts of interest.

## Supporting information


**Table S1.** List of CLPB variants reported so far in CLPB‐deficient patients.
**Table S2.** Effects of CLPB disruption on cell proteome.
**Table S3.** CLPB interactome.
