# Supplementary material for: CLPB Deficiency, a Mitochondrial Chaperonopathy With Neutropenia and Neurological Presentation
Source: J Inherit Metab Dis. 2025 Apr 7;48(3):e70025. doi: 10.1002/jimd.70025 (PMC11975511; doi:10.1002/jimd.70025)
Supplement: Supplementary file 1 — Table S1. List of CLPB variants reported so far in CLPB‐deficient patients. Table S2. Effects of CLPB disruption on cell proteome. Table S3. CLPB interactome. [file JIMD-48-0-s001.docx]

SUPPL. TABLE 1

List of CLPB variants reported so far in *CLPB*-deficient patients

| DNA sequence variant NM_030813.6 | Pathogenic protein variant  NP_110440.1 | Type of variant | Mode of inheritance | Presence in individuals | Reported in | Protein region | Characterised on protein level | ATPase activity | Disaggre-gase activity | Negative effect on wild-type subunit ATPase | Negative effect on wild-type subunit disaggre-gation | Characterised in cells |
| --- | --- | --- | --- | --- | --- | --- | --- | --- | --- | --- | --- | --- |
| c.216del | R73Afs* 168 | frameshift | recessive | in *trans* with R408W (one individual, moderate phenotype) | Pronicka et al 2017 |  | NO | N/A | N/A | N/A | N/A | NO |
| c.491A>C | H164P | missense | recessive | In *trans* with A359_A360insDLVCLAV (two individuals, severe phenotype in one, n.d. for the other) | Farrow et al, 2023 | ankyrin region – between ankyrin 1 and 2 | NO | N/A | N/A | N/A | N/A | NO |
| c.748C>T | R250* | nonsense | recessive | together with E501K, in *trans* with R417* (two individuals, severe phenotype) | Wortmann et al., 2015 |  | NO | N/A | N/A | N/A | N/A | NO |
| c.803C>T | **T268M** | missense | recessive | homozygous (four individuals, moderate phenotype)  Founder variant in Inuit of Greenland | Saunders et al., 2015; | ankyrin repeat 3 | ^PARL^CLPB  increased ATPase actiivity (Cupo and Shor-ter, 2020)  ~30%  disaggregase^luc^ (Cupo and Shorter 2020; Gupta et al., 2023; Lee et al., 2023),  impaired disaggrega-se^luc, syn^ in oxi-dative environment (Lee et al., 2023)  Normal interaction with HAX1 (Fan et al., 2022) | + | - | N/A | N/A | Normal activity of ATP synthase in patient fibro-blasts (Saunders et al., 2015) |
| c.805G>A | A269T | missense | recessive | In *trans* with Y272C (one individual, moderate phenotype) | Kiykim et al., 2016; | ankyrin repeat 3 | ^PARL^CLPB  Severely impaired dis-agreggase^luc^ activity (Gupta et al., 2023; Lee et al., 2023)  slightly impaired dis-aggregase^syn^  activity | + | - | N/A | N/A | NO |
| c.815A>G | Y272C | missense | recessive | In *trans* with Y567C (two individuals, severe phenotype) | Wortmann et al., 2015 | ankyrin repeat 3 | ^PARL^CLPB  Disaggregase activity  severely impaired dis-agreeggase^luc^ activity (Gupta et al., 2023)  slightly decreased dis-aggregase^luc^ activity (Lee et al., 2023)  increased disaggre-gase^syn^ activity severely impaired disaggrega-se^luc, syn^ in oxidative en-vironment (Lee et al., 2023)  disrupted interaction with HAX1 (Fan et al, 2022) | - | - | N/A | N/A | Lentiviral expression in CLPB^KO^ Hap1 cells (Lee et al., 2023)  - lack of recovery of insoluble pro-teins  - further decrease of solubility of HAX1 (less soluble than in knock-out cells) |
|  |  |  |  | In *trans* with A269T (one individual, moderate phenotype) | Kiykim et al., 2016 |  |  |  |  |  |  |  |
| c.961A>T | K321* | nonsense | recessive | in *trans* with R417* (one individual, severe phenotype) | Saunders et al., 2015 |  |  |  |  | N/A |  | in *trans* with R417*  analysis of res-piration in patient liver  normal activity of complexes I and IIl  severely reduced activity of com-plex III  in-gel assay: nor-mal activity of complexes I and IV |
| c.1079-23T>A | A359_A360 ins DLVCLAV | splicing error: insertion | recessive | in *trans* with H164P (two individuals, severe phenotype in one, n.d. for the other) | Farrow et al., 2023 | insertion in P-loop | NO | N/A | N/A | N/A | N/A | NO |
| c.1159C | **R387*** | nonsense | recessive | homozygous (two individuals, severe phenotype) | Darouich et al. 2023 |  | NO | N/A | N/A | N/A | N/A | NO |
| c. 1163C>A | T388K | missense | dominant | monoallelic (one individual) | Warren et al., 2022; Tucker et al., 2022^3^ | AAA+ module, direct neighbourhood of Walker A motif | NO | N/A | N/A | N/A | N/A | NO |
| c.1211A>C | K404T | missense | dominant | monoallelic (one individual) | Wortmann et al., 2021 | AAA+ module, interface between protomers | ^MPP^CLPB  ATPase, disaggregase – alone and in 1:1 mix with wt (Wortmann et al., 2021) | - (40%) | - | N/A | YES | NO |
| c.1222A>G | R408G | missense | recessive | In *trans* with R417* (four individuals, moderate phenotype) | Wortmann et al.,2015; Pronicka et al., 2017 | AAA+ module, interface between protomers (clockwise | ^MPP^CLPB  ATPase activity – 20% of wild-type (Wortmann et al., 2015)  ^PARL^CLPB  ATPase, disaggregase – alone and in 1:1 mix with wt (Warren et al., 2022) | - (20%) | - (0) | NO | NO | Lentiviral over- expression in MOLM-13 cells (Warren et al., 2022):  - normal basal and maximal respiration  - normal ATP production |
|  |  |  |  | in *trans* with D462R*11 (one individual, moderate phenotype)  iIn *trans* with R73Afs*168 (one individual, moderate phenotype) | Pronicka et al., 2017  Pronicka et al., 2017 |  |  |  |  |  |  |  |
| c.1233G>A | M411I | missense | recessive | in *trans* with Y617C (two individuals, mild phenotype) | Wortmann et al. 2015; | AAA+ mo-dule, folded core of the large sub-unit of the ATPase | NO | N/A | N/A | N/A | N/A | NO |
| c.1249C>T | **R417*** | nonsense | recessive | in *trans* with R408G (four individuals, moderate phenotype) | Wortmann et al.,2015;  Pronicka et al., 2017^,^ |  | NO | N/A | N/A | N/A | N/A |  |
| *(cont.)* |  |  |  | in t*rans* with K321* (one individual,  severe phenotype) | Saunders et al., 2015 |  |  |  |  |  |  | in *trans* with K321*  analysis of respi-ration in patient liver  normal activity of complexes I and IIl  severely reduced activity of complex III  in-gel assay: normal activity of complexes I and IV |
|  |  |  |  | in *trans* with R250* in together with E501K (two individuals, severe phenotype) | Wortmann et al.2015; |  |  |  |  |  |  |  |
|  |  |  |  | homozygous (one individual, severe phenotype) | Pronicka et al., 2017 |  |  |  |  |  |  |  |
|  |  |  |  | in *trans* with 374_389del (two individuals, mild phenotype) | Tucker et al., 2022 |  |  |  |  |  |  |  |
| c.1257+5 G>A | 374_389del | splicing error: (exon 11 skipping) | recessive | in *trans* with R417* (one individual, mild phenotype) | Tucker et al., 2022 | deletion of 15 amino acids between Walker A and pore loop | NO | N/A | N/A | N/A | N/A | patient lymphoblasts (in *trans* with R417*)  changes in protein abundance in patient lymphoblasts (detailed jn Suppl. Table 2) |
| c.1257+5G>T | p.? | splicing error | recessive | in *trans* with R417* (one individual, mild phenotype) | Tucker et al., 2022 | ? (predicted similar to c.1257+5 G>A) | NO | N/A | N/A | N/A | N/A | NO |
| c.1280C>T  *(cont.)* | P427L | missense | dominant | monoallelic (one individual) | Wortmann et al., 2021 | AAA+ module, immediate vicinity of pore loop 1 | YES  ^MPP^CLPB  ATPase, disaggregase – alone and in 1:1 mix with wt (Wortmann et al., 2021) | - (60%) | - (0) | N/A | YES | complexome (Wortmann et al., 2021)  increased total abundance of CLPB, main peak 1.3 MDa  changes in abundance and migration patterns of mitochondrial proteins (described in more detail in Suppl. Table 2) |
| c.1305_1307 delinsCCC | E435_G436 delinsDP | missense | recessive | In *trans* with G646V (one individual, moderate phenotype) | Wortmann et al. 2015;  Tucker et al., 2022^1^ | AAA+ module, immediate vicinity of pore loop 1 | NO | N/A | N/A | N/A | N/A | patient fibroblasts (in trans with G646V)    normal OXPHOS activity in patient fibroblasts (Wortmann et al., 2015) complexome (Wortmann et al., 2015 and 2021) |
| c.1383dupA | D462R*11 | frameshift | recessive | in *trans* with R408G, (one individual, moderate phenotype) | Pronicka et al. 2017 |  | NO | N/A | N/A | N/A | N/A | NO |
| c.1424G>A | **R475Q** | missense | recessive | homozygous (one individual, severe phenotype) | Pronicka et al., 2017 | AAA+ module, interface between protomers | ATPase, disaggregase^luc^ (Cupo and Shorter 2020) | - | - | N/A | N/A | NO |
| c.1456T>C | **C486R** | missense | recessive | homozygous (two individuals, moderate phenotype) | Wortmann et al. 2015; | AAA+ module, folded core of the large subunit of the ATPase | NO | N/A | N/A | N/A | N/A | NO |
| c.1488T>A  *(cont.)* | N496K | missense | dominant | monoallelic (one individual) | Warren et al., 2022 | AAA+ module, sensor-1 motif | YES  ^PARL^CLPB  ATPase, disaggregase^luc^ – alone and in 1:1 mix with wt (Warren et al., 2022) | - | - (0) | YES | YES | YES  Lentiviral expression in cord blood CD341 cells (Warren et al., 2022):  -decreased CFU-G  - decrease in neutrophils  - increase in granulocytic pre-cursors  Lentiviral overexpression in MOLM-13 cells (Warren et al., 2022):  - reduced basal and maximal res-piration  - reduced ATP production |
| c.1669G>A | E557K | missense | dominant | monoallelic (one individual) | Warren et al., 2022 | AAA+ module in the neigh-bourhood of the arginine finger | YES  ^PARL^CLPB  ATPase, disaggregase – alone and in 1:1 mix with wt (Warren et al., 2022) | - (0) | - (0) | YES | YES | YES  Lentiviral expression in cord blood CD341 cells (Warren et al., 2022):  -decreased CFU-G  - decrease in neutrophils  - increase in granulocytic precursors  Lentiviral overexpression in MOLM-13 cells (Warren et al., 2022):  - reduced basal and maximal respiration  - reduced ATP production |
| c.1678G>A  *(cont.)* | G560R | missense | dominant | monoallelic (three individuals) | Wortmann et al., 2021  Fan et al., 2022 | AAA+ module in the neighbourhood of the arginine finger | YES  ^MPP^CLPB  ATPase, disaggregase^luc^ – alone and in 1:1 mix with wt (Wortmann et al., 2021) | - (10%) | - (0) | N/A | YES | patient-derived fibroblasts: complexome (Wortmann et al., 2021)  -decreased total abundance of CLPB  Only peak at 2.1 MDa (no 1.3 MDa peak)  changes in abun-dance and mig-ration patterns of mitochondrial proteins (described in more detail in Table 2) |
| c.1681C>G | R561G | missense | dominant | monoallelic (one individual) | Warren et al. 2022 | AAA+ module, arginine finger | YES  ^PARL^CLPB  ATPase, disaggregase^luc^ – alone and in 1:1 mix with wt (Warren et al., 2022) | - (0) | - (0) | YES | YES | YES  Lentiviral expression in cord blood CD341 cells:  -decreased CFU-G  - decrease in neutrophils  - increase in granulocytic pre-cursors  Lentiviral overexpression in MOLM-13 cells (Warren et al., 2022):  - reduced basal and maximal res-piration  - reduced ATP production |
| c.1681C>T  *(cont.)* | R561W | missense | dominant | monoallelic (two individuals) | Wortmann et al., 2021 | AAA+ module, arginine finger | NO | N/A | N/A | N/A | N/A | patient-derived fibroblasts: complexome (Wortmann et al., 2021)  increased total abundance of CLPB, main peak 1.3 MDa  changes in abundance and migration patterns of mitochondrial proteins (described in more detail in Suppl. Table 2) |
| c.1682G>A | R561Q | missense | dominant | monoallelic (six individuals) | Warren et al., 2022  Fan et al., 2022;  Tucker et al., 2022^1^ | AAA+ module, arginine finger | YES  ^PARL^CLPB  intact interaction with HAX1 (Fan et al., 2022) | N/A | N/A | N/A | N/A | YES  Lentiviral overexpression in MOLM-13 cells (Warren et al., 2022):  - reduced basal and maximal respiration  - reduced ATP production |
| c.1685delT | **I562Tfs*23** | frameshift | recessive | homozygous (four individuals, severe phenotype) | Capo-Chichi et al., 2015 |  | NO | N/A | N/A | N/A | N/A | NO |
| c.1700A>G | Y567C | missense | recessive | in *trans* with Y272C (two individuals, severe phenotype) | Wortmann et al. 2015; | AAA+ module, folded core of the large subunit of the ATPase | NO | N/A | N/A | N/A | N/A | NO |
| c.1772C>T | **A591V** | missense | recessive | homozygous (one individual, severe phenotype) | Wortmann et al. 2015; | AAA+ module – stabilisation of the fol-ding of the small sub-unit of the ATPase | YES  ^PARL^CLPB  no ATPase and disaggre-gase activity (Cupo and Shorter 2020) | - (0) | - (0) | N/A | N/A | homozygous patient  normal OXPHOS activity in patient fibroblasts (Wortmann et al., 2015) complexome (Wortmann et al., 2015 and 2021) |
| c.1850A>G | Y617C | missense | recessive | in *trans* with M411I (two individuals, mild phenotype) | Wortmann et al. 2015; | AAA+ module, interface between protomers | NO | N/A | N/A | N/A | N/A | NO |
| c.1858C>T  *(cont.)* | R620C | missense | dominant | monoallelic (two individuals) | Warren et al., 2022 | AAA+ module sensor-2 motif | YES  ^PARL^CLPB  ATPase, disaggregase – alone and in 1:1 mix with wt (Warren et al., 2022) | - (0) | - (0) | YES | YES | YES  Lentiviral expression in cord blood CD341 cells:  -decreased CFU-G  - decrease in neutrophils  - increase in granulocytic precursors  Lentiviral overexpression in MOLM-13 cells (Warren et al., 2022):  - reduced basal and maximal respiration  - reduced ATP production |
| c.1859G>A | 620H | missense | dominant | monoallelic (one individual) | Fan et al., 2022 | AAA+ module sensor-2 motif | NO | N/A | N/A | N/A | N/A | NO |
| c.1882C>T | R628C | missense | recessive | In *trans* with E639K (two individuals, moderate phenotype) | Kanabus et al. 2015;  Tucker et al. 2022^1^ | AAA+ module, interface between protomers | NO | N/A | N/A | N/A | N/A | NO |
|  |  |  |  | In *trans* with A635 (one individual, severity – not provided) | Rivalta et al., 2022 |  |  |  |  |  |  |  |
| c.1903_1904delinsAA | A635K | missense | recessive | In *trans* with R628C (one individual, severity – not provided) | Rivalta et al., 2022 | AAA+ module, interface between protomers | NO | N/A | N/A | N/A | N/A | NO |
| c.1915G>A | E639K | missense | recessive | In *trans* with R628C (two individuals, moderate phenotype) | Kanabus et al. 2015  Tucker et al. 2022^1^ | AAA+ module, interface between protomers | NO | N/A | N/A | N/A | N/A | NO |
| c.1937dupG | C647Lfs*26 | frameshift | recessive | in *trans* with I682N (one individual, severe phenotype) | Wortmann et al. 2015 |  | NO | N/A | N/A | N/A | N/A | NO |
| c.1937G>T | G646V | missense | recessive | in *trans* with E435_G436 delinsDP (one individual) | Wortmann et al.,2015;  Tucker et al., 2022^1^ | AAA+ module - stabilisation of the folding of the small subunit of the ATPase | NO | N/A | N/A | N/A | N/A | patient fibroblasts (in *trans* with E435_G436 delinsDP)    normal OXPHOS activity in patient fibroblasts (Wortmann et al., 2015) complexome (Wortmann et al., 2015 and 2021) |
| c.1949G>C | **R650P** | missense | recessive | homozygous (one individual, severe phenotype) | Pronicka et al., 2017 | AAA+ module - stabilisation of the folding of the small subunit of the ATPase | YES  ^PARL^CLPB  wild-type ATPase activity  no disaggregase^luc^ activity (Cupo and Shorter 2020) | WT | - (0) | N/A | N/A | NO |
| c.2045T>A | I682N | missense | recessive | in *trans* with C647Lfs*26 (one individual, severe phenotype) | Wortmann et al. 2015; | C-terminal region - stabilisation of the folding of the small subunit of the ATPase | NO | N/A | N/A | N/A | N/A | NO |

in bold: recessive variants homozygous in individuals

shaded: analysed biochemically or in cells

^1^new data on previously reported individuals

^luc^firefly luciferase as substrate

^syn^α-synuclein-GFP as substrate

SUPPL. TABLE 2

Effects of CLPB disruption on cell proteome

| Described in | Cells | Issue | Effects | Processes |
| --- | --- | --- | --- | --- |
| Cupo and Shorter 2020 | Hap1, CLPB KO | Protein solubility | Decreased solubility:  HAX1 | Regulation of apoptosis, cell migration, granulopoiesis |
|  |  |  | PARL | Regulation of apoptosis, proteolysis |
|  |  |  | OPA1 | Regulation of apoptosis, maintenance of cristae structure |
|  |  |  | PHB2 | Regulation of apoptosis, regulation of proteolysis, maintenance of supercomplexes in IMM |
|  |  |  | HTRA2 | Regulation of apoptosis, proteolysis |
|  |  |  | SMAC/DIABLO | Apoptosis |
|  |  |  | SLC proteins including  SLC25A13 (ARL2), SLC25A25, SLC25A32 (MTF),  SLC25A33, SLC25A36, SLC25A39, SLC25A4 (ANT1), SLC25A5 (ANT2),  SLC25A6 (ANT3), SLC25A12 (ARL1). | Small molecule transport: mitochondrial solute carriers |
|  |  |  | MICU1, MICU2 | Calcium import to mitochondria |
|  |  |  | TIMM8A, TIMM8B, TIMM13, TIMM21, TIMM22, TIMM23, TIMM50 | Transport to mitochondria (inner membrane translocase) |
|  |  |  | NDUFA8, NDUFA11, NDUFA13, NDUFB7, NDUFB10, TTC19, COX11, CYC1 | OXPHOS |
| Fan et al. 2022 | PLB-985, CLPB KO | Mitochondrial protein concentration | Significantly increased protein concentration |  |
|  |  | Mitochondrial protein synthesis and persistence | Increased synthesis (slightly, increased persistence, not statistically significant)  NDUFA9, NDUFA7, NDUFA12, NDUFB8, NDUFB10, NDUFB9, NDUFS2, NDUFA6, NDUFA11, NDUFA13, NDUFA2, NDUFA10 | OXPHOS – complex I |
|  |  |  | Increased synthesis (slightly, increased persistence, not statistically significant)  UQCRC1, UQCRC2, UQCRQ | OXPHOS – complex III |
|  |  |  | Increased synthesis (slightly, increased persistence, not statistically significant)  COX4I1, COX5A, MT-CO3 | OXPHOS – complex IV |
|  |  |  | Increased synthesis  ATP5C1 | OXPHOS – complex V |
|  |  |  | Increased synthesis  SUCLG1, DLST, FH, ACO2, DLAT, SUCLG2, NNT, OGDH, CS, IDH3A, SDHA, PDHA1, PDHB, DLD, IDH3B, MDH1 | TCA cycle |
| Baker et al. 2024 | Flp-In T-REx 293 (HEK293T) CLPB KO | Protein solubility:  +/- heat shock  +/- reintroduction of extrageneous CLPB-FLAG  ^1^insoluble in CLPB KO  ^2^insoluble in CLPB KO after reintroduction of extrageneous CLPB-FLAG  ^3^ insoluble in CLPB KO after heat shock  ^4^ insoluble in CLPB KO after heat shock and reintroduction of extrageneous CLPB-FLAG | AFG3L2^1,3^, YME1L1^3^ | proteolysis |
|  |  |  | CPOX^3^ | Haem biosynthesis |
|  |  |  | CYC1^3^, MT-CO2^1,3,4^, NDUFA13^1,3,4^ | OXPHOS |
|  |  |  | GHITM^3^ | Mitochondrial morphology, calcium & potassium homeostasis, apoptosis |
|  |  |  | GPD2^3^ | Glycerol metabolism |
|  |  |  | HAX1^1,2,3,4^ | Regulation of apoptosis, cell migration, granulopoiesis |
|  |  |  | HTRA2^3^ | Regulation of apoptosis, proteolysis |
|  |  |  | IMMT^1^ | Mitochondrial membrane organisation – MICOS complex |
|  |  |  | MICU2^3^ | Calcium import to mitochondria |
|  |  |  | NNT^1,2^ | Redox homeostasis |
|  |  |  | OPA1^1,3,4^ | Regulation of apoptosis, maintenance of cristae structure |
|  |  |  | SAMM50^1^ | Protein import and sorting: SAM complex; Mitochondrial membrane organisation – MICOS complex |
|  |  |  | SLC25A10^3,4^, SLC25A13^3,4^ | Small molecule transport: mitochondrial solute carriers |
|  |  |  | STOML2^1,3,4^ | Protein homeostasis, stabilisation of protein supercomplexes |
|  |  |  | TIMM13^3^ | Chaperone, protein import and sorting |
|  |  |  | TIMM50^3^ | Protein import into mitochondria: TIM complex |
|  |  | Changes in protein abundance | Increased abundance |  |
|  |  |  | BAX, ENDOG | apoptosis |
|  |  |  | ACOT7, FASN , PAICS , PCK2 | Cellular metabolism |
|  |  |  | PABPN1, PAK4 | Signalling |
|  |  |  | PRDX4, TXNRD1 | Redox homeostasis |
|  |  |  | FKBP8 | Regulation of apoptosis, autophagy, mitophagy |
|  |  |  | RAB11B | Protein & anion transport, exocytosis |
|  |  |  | Decreased abundance |  |
|  |  |  | ELAC2, KIAA0391, MRPS12, TEFM | Mitochondrial transcription, translation and RNA processing |
|  |  |  | FAHD1, GK | Cellular metabolism |
|  |  |  | COX6C, FOXRED1, SCO1 | OXPHOS |
|  |  |  | HSCB (HSC20) | Fe-S cluster biosynthesis |
|  |  |  | OMA1 | Proteolysis, apoptosis, quality control, cristae maintenance |
| Wortmann et al., 2021  *(cont.)* | Patient fibroblasts  patients with monoallelic CLPB defect – P427L; G560R; R561W  Patients with biallelic CLPB defect:  homozygous: p. A591V  comp. heterozygous E435D, G436P + p.G646V | Changes in protein abundance in mitochondria-enriched fractions | Biallelic:  Increased abundance: 54 proteins  decreased abundance: 43 proteins |  |
|  |  |  | Monoallelic:  Increased abundance: 147 proteins  Decreased abundance: 98 proteins | - cell adhesion and projection  - neuronal development  - regulation  of apoptosis  - stabilization of membrane curvature  - membrane-cytoskeleton  interaction |
|  |  | Changes in mitochondrial complexome caused by monoallelic *clpb* mutations (migration patterns in hrCN-PAGE [high-resolution clear native PAGE]) | Aberrant migration patterns (complex size)  Smaller complexes: |  |
|  |  |  | MAVS | Immune response (RIG-I pathway) |
|  |  |  | TOMM70 | Transport into mitochondria (TOM complex) |
|  |  |  | CLPX | proteolysis |
|  |  |  | ACADVL | fatty acid beta-oxidation |
|  |  |  | FKBP8 | Regulation of apoptosis, autophagy, mitophagy |
|  |  |  | TUFM | mitochondrial protein synthesis, autophagy, immune response |
|  |  |  | AHNAK | Neuronal differentiation |
|  |  |  | PDCD6IP | i.a. apoptosis, cytokinesis, exosome biogenesis and excretion |
|  |  |  | Larger complexes: |  |
|  |  |  | HAX1 | Regulation of apoptosis, cell migration, granulopoiesis |
|  |  |  | HIKESHI | Response to heat stress, protein import to nucleus |
|  |  |  | DCHR24 | cholesterol synthesis, apoptosis |
|  |  |  | MFN2 | mitochondrial fusion |
|  |  |  | AIFM1 | Regulation of apoptosis, OXPHOS assembly, protein import into mitochondria |
|  |  |  | RAI14 | Early morphogenesis of neurons |
|  |  |  | KIDINS220 | Neuronal development |
|  |  |  | KANK2 | regulation of transcription, regulation of apoptosis, signalling, cell motility |
| Tucker et al., 2022 | Patient lymphoblasts – patient with recessive CLPB deficiency: p.417 + 374_389del (resulting from a splicing error leading to exon 11 skipping) | Total level of CLPB | Significantly decreased total level of CLPB |  |
|  |  | Changes in protein abundance | Increased abundance |  |
|  |  |  | IGLL5, FCRLA, MNDA | B-cell differentiation and receptor signalling |
|  |  |  | TCL1A | Cell proliferation and survival, stabilisation of IMM potential |
|  |  |  | ENDOG, BAK1, HAX1 | apoptosis |
|  |  |  | Decreased abundance |  |
|  |  |  | PFN2, MICAL3, CNN3, INPP5F | Actin and cytoskeleton modification, cell migration |
|  |  |  | ACAD5, FDXR, APOL2, DHRS1, AMACR | Lipid metabolism |
|  |  |  | No reduction | OXPHOS |

Changes in the cellular or mitochondrial proteome driven by CLPB knock-out in commercial cell lines or pathogenic variants in patient-derived cells. Functional annotation of the proteins is based on the data provided by the authors of the articles in question, GO biological process annotation, Uniprot description, and for mitochondrial proteins – additionally MitoCarta 3.0.

SUPPLEMENTARY TABLE 3

CLPB interactome

| Protein | Function | Related pathologies |
| --- | --- | --- |
| HAX1 | HCLS1-associated protein; regulatory protein which cellular localisation includes the endoplasmic reticulum, nucleus and mitochondrial intermembrane space (Suzuki et al. 1997). Has antiapoptotic activity (Chao et al. 2008, Yan et al. 2015), evident in various tumours, where *HAX1* overexpression is correlated with disease progression and poor prognosis (Trebińska et al. 2010, Yap et al. 2011).  Has been implicated to participate in a plethora of cellular functions, such as cell migration, cytoskeletal regulation, calcium homeostasis (Vafiadaki et al. 2009, Hirasaka et al. 2016, Larsen et al. 2020), and RNA processing.  HAX1 mutations underlie a clinical entity known as Kostmann syndrome Its main manifestation is severe congenital neutropenia with occasional neurological symptoms. Due to the partial overlap with the symptoms of CLPB mutations, a link between the two proteins has been long postulated (Wortmann et al. 2015). | Severe Congenital Neutropenia type 3 (MIM **#** 610738)  (Kostmann syndrome) |
| HTRA2 | Serine protease localised to the mitochondrial intermembrane space. Participate in apoptosis (Suzuki et al. 2001). HAX1 present HTRA2 to the PARL protease to activate it (Chao et al.2008) | *Parkinson disease 13 (MIM 610297): conflicting data*  3-methylglutaconic aciduria typ VIII (MIM 617248) |
| MAVS | Mitochondiral antiviral signaling protein, participating in innate antioviral immunity. Act as intermediate in NF-kappaB, IRF3 and IRF7 activation in response to detection of viral ds-DNA (Seth et al. 2005). | (-) |
| OPA1 | Optic atrophy protein 1 (OPA1), a dynamin-related GTPase is a key component of mitochondrial cristae (Chen et al. 2019). It is located either in the inner mitochondrial membrane, mostly cristae membrane facing the mitochondrial intermembrane space, or, in its cleaved form, in the IMS.  Its functions include driving mitochondrial fusion, maintaining cristae structure, controlling cytochrome c release (Frezza et al. 2006), safeguarding mitochondrial DNA (Elachouri et al. 2011), ensuring the activity of the oxidative phosphorylation system (Yoshizumi et al. 2017) and preventing apoptosis  CLPB ablation results in an overall increase in the level of OPA1 and accumulation of S-OPA1 (Chen et al. 2019). A direct interaction between the two proteins is suggested by co-immunoprecipitation. Chen et al. (2019) additionally posit that CLPB and OPA1 cooperate in the process of cristae maintenance, based on the similarity in mitochondrial morphology after deletion of either protein. | Optic atrophy 1  (MIM 165500)  Behr syndrome  (MIM 210000)  Optic atrophy plus syndrome  (MIM 125250) |
| PARL | Presenilin-associated rhomboid-like protein, a protease located in the inner mitochondrial membrane (Greene 2012). Forms SPY complexes with STOML2 and YME1L1, which regulates the activity of both proteases activity of its components (Wai et al. 2016, Baker et al. 2024). CLPB has been recently shown to associate with other members of the SPY complex, as well as contribute to their solubility, stability and activity (Baker et al. 2024). | (-) |
| PHB1/PHB2 | Prohibitins, multifunctional proteins participating in cell signalling, chaperone and transciptional regulation roles.  Prohibitins form multimeric ring-shaped structures primarily anchored in the inner mitochondrial membrane (IMM), consisting of two interdependent types of subunits, PHB1 and PHB2. The loss of PHB subunits results in abnormal cristae morphology, inhibition of mitochondrial fusion, fragmentation of the mitochondrial network, and increased susceptibility to aptoptotic stimuli (Merkwirth et al. 2008, Sato et al. 2011).  Form a mutually stabilising complex with STOML2 (Da Cruz et al. 2008), creating a cardiolipin-rich microenvironment, which might contribute to formation or stability of respiratory supercomplexes (Acín-Pérez et al. 2008, Christie et al. 2011, Christie et al. 2012, Mitsopoulos et al. 2015, Jian et al. 2017).  In association with DNAJC19 control cardiolipin remodelling  Form a supercomplex with DNAJC19 and the m-AAA protease (Richter-Dennerlein et al. 2014).  Interact with the i-AAA protease and OMA1 to regulate OPA1 processing (Richter-Dennerlein et al. 2014). | *susceptibility to breast cancer (MIM114480): conflicting data* |
| STOML2 | Stomatin-like protein 2, regulare biogenesis and activity of mitochondria. (Seth et al. 2005)  Forms SPY complexes with PARL and YME1L1, which regulates the activity of both proteases (Baker et al. 2024)  Forms mutually stabilising complexes with prohibitins PHB1/PHB2 (Seth et al. 2005). | (-) |
| YME1L1  (i-AAA+ protease) | Mitochondrial escape 1-like, an ATP-dependent metalloprotease of AAA family (Wai et al.2016) .  Forms SPY complexes with STOML2 and PARL, which regulates the activity of both proteases (Baker et al. 2024)  Participate in proteolytic processing of OPA1 (Elachouri et al.2011) | Optic atrophy 11  (MIM 617302) |

Supplementary bibliography

Acín-Pérez R, Fernández-Silva P, Peleato ML, Pérez-Martos A, Enriquez JA. Respiratory active mitochondrial supercomplexes. *Mol cell*. 2008;*32*(4):529–539

Baker MJ, Blau KU, Anderson AJ, et al.. CLPB disaggregase dysfunction impacts the functional integrity of the proteolytic SPY complex. *J cell biol.* 2024;223(3):e202305087.

Capo-Chichi JM, Boissel S, Brustein E, et al. Disruption of CLPB is associated with congenital microcephaly, severe encephalopathy and 3-methylglutaconic aciduria. *J med genet.* 2015*;* 52(5):303-311.

Chao JR, Parganas E, Boyd K, Hong CY, Opferman JT, Ihle JN. Hax1-mediated processing of HtrA2 by Parl allows survival of lymphocytes and neurons. *Nature*, 2008;452(7183):98-102

Chen X, Glytsou C, Zhou H, et al. Targeting Mitochondrial Structure Sensitizes Acute Myeloid Leukemia to Venetoclax Treatment. *Cancer discov*. 2019;*9*(7):890–909.

Christie DA, Lemke CD, Elias IM, et al. Stomatin-like protein 2 binds cardiolipin and regulates mitochondrial biogenesis and function. *Mol cell biol*. 2011;*31*(18):3845–3856

Christie DA, Mitsopoulos P, Blagih J, et al. Stomatin-like protein 2 deficiency in T cells is associated with altered mitochondrial respiration and defective CD4+ T cell responses. J *immunol*. 2012;*189*(9):4349-4360

Cupo RR, Shorter J. Skd3 (human ClpB) is a potent mitochondrial protein disaggregase that is inactivated by 3-methylglutaconic aciduria-linked mutations. *Elife,* 2020;*9*:e55279.

Da Cruz S, Parone PA, Gonzalo P, et al. SLP-2 interacts with prohibitins in the mitochondrial inner membrane and contributes to their stability. *Biochim biophys acta*, 2008;*1783*(5):904–911

Darouich S, Darouic, S, Gtari D, Bellamine H. CLPB Deficiency Associated Neonatal Cavitating Leukoencephalopathy: A Potential Pathomechanism Underlying Neurologic Disorder. *Pediatr dev pathol.* 2023;27(2):198–204.

Elachouri G, Vidoni S, Zanna C, et al. OPA1 links human mitochondrial genome maintenance to mtDNA replication and distribution. *Genome res*. 2011 Jan;21(1):12-20.

Fan Y, Murgia M, Linder MI, et al. HAX1-dependent control of mitochondrial proteostasis governs neutrophil granulocyte differentiation. *J clin investig* 2022;132(9):.e153153.

Farrow EG, Jay A, Means JC, et al. Case of CLPB deficiency solved by HiFi long read genome sequencing and RNAseq. *Am j med genet A*, 2023;191(12);2908.

Frezza C, Cipolat S, Martins de Brito O, et al. OPA1 controls apoptotic cristae remodeling independently from mitochondrial fusion. *Cell*. 2006;126(1):177–189.

Greene AW, Grenier K, Aguileta MA, et al. Mitochondrial processing peptidase regulates PINK1 processing, import and Parkin recruitment. *EMBO rep*. 2012;13(4):378-85

Gupta A, Lentzsch AM, Siegel A, et al. Dodecamer assembly of a metazoan AAA+ chaperone couples substrate extraction to refolding. Sci *adv.* 2023*;9*(19):eadf5336.

Hirasaka, K., Mills, E. M., Haruna, M., et al. UCP3 is associated with Hax-1 in mitochondria in the presence of calcium ion. *Biochem biophys res commun.* 2016;472(1):108–113

Jian, C., Xu, F., Hou, T., Sun, T., Li, J., Cheng, H., & Wang, X. (2017). Deficiency of PHB complex impairs respiratory supercomplex formation and activates mitochondrial flashes. *J cell sci*, *130*(15), 2620-2630.

Kanabus, M, Shahni R, Saldanha JW, et al. Bi-allelic CLPB mutations cause cataract, renal cysts, nephrocalcinosis and 3-methylglutaconic aciduria, a novel disorder of mitochondrial protein disaggregation. *J inherit metab dis.* 2015;38: 211-219.

Kiykim A, Garncarz W, Karakoc-Aydiner E, et al. Novel CLPB mutation in a patient with 3-methylglutaconic aciduria causing severe neurological involvement and congenital neutropenia. *Clin immunol.* 2016;100(165):1-3.

Lee S, Lee SB, Sung N, et al.. Structural basis of impaired disaggregase function in the oxidation-sensitive SKD3 mutant causing 3-methylglutaconic aciduria. *Nat comm.* 2023;*14*(1):2028.

Larsen EK, Weber DK, Wang S, et al. Intrinsically disordered HAX-1 regulates Ca^2+^ cycling by interacting with lipid membranes and the phospholamban cytoplasmic region. *Biochim biophys acta. Biomembranes*. 2020;*1862*(1):183034.

Merkwirth C, Dargazanli S, Tatsuta T, et al. (2008). Prohibitins control cell proliferation and apoptosis by regulating OPA1-dependent cristae morphogenesis in mitochondria. *Genes dev.* *22*(4), 476–488.

Mitsopoulos P, Chang YH, Wai T, et al.). Stomatin-like protein 2 is required for in vivo mitochondrial respiratory chain supercomplex formation and optimal cell function. *Mol cell biol.* 2015;*35*(10):1838-1847.

Pronicka E, Ropacka‐Lesiak M, Trubicka J, et al. A scoring system predicting the clinical course of CLPB defect based on the foetal and neonatal presentation of 31 patients. *J inherit metab dis* 2017;40(6):853-860.

Richter-Dennerlein R, Korwitz A, Haag M, et al. DNAJC19, a mitochondrial cochaperone associated with cardiomyopathy, forms a complex with prohibitins to regulate cardiolipin remodeling. *Cell metab*. 2014;*20*(1):158-171

Rivalta B, Torraco A, Martinelli D, Luciani M, Carrozzo R, Finocchi A. Biallelic CLPB mutation associated with isolated neutropenia and 3‐MGA‐uria. *Pediatr allergy immunol.* 2022;33(5): e13782

Sato SI, Murata A, Orihara T, et al. Marine natural product aurilide activates the OPA1-mediated apoptosis by binding to prohibitin. *Chem biol.* 2011;*18(*1):131-139

Saunders C, Smith L, Wibrand F, et al. CLPB variants associated with autosomal-recessive mitochondrial disorder with cataract, neutropenia, epilepsy, and methylglutaconic aciduria. *Am j hum genet*. 2015; 96(2):258-265.

Seth RB, Sun L, Ea CK, Chen ZJ. Identification and characterization of MAVS, a mitochondrial antiviral signaling protein that activates NF-kappaB and IRF 3. *Cell*. 2005 Sep 9;122(5):669-82

Suzuki Y, Demoliere C, Kitamura D, Takeshita H, Deuschle U, Watanabe T. HAX-1, a novel intracellular protein, localized on mitochondria, directly associates with HS1, a substrate of Src family tyrosine kinases. *J immunol (Baltimore)*, 1997;*158*(6), 2736–2744.

Suzuki Y, Imai Y, Nakayama H, Takahashi K, Takio K, Takahashi R. A serine protease, HtrA2, is released from the mitochondria and interacts with XIAP, inducing cell death. *Molec cell.* 2001;8:613-621

Trebinska A, Rembiszewska A, Ciosek K, et al. HAX-1 overexpression, splicing and cellular localization in tumors. *BMC cancer*. 2010;*10*:76

Tucker EJ, Baker MJ, Hock DH, et al.. Premature ovarian insufficiency in CLPB deficiency: Transcriptomic, proteomic and phenotypic insights. *J clin endocrinol metab.* 2022;107*(*12):3328-3340.

Vafiadaki E, Arvanitis DA, Pagakis SN, et al. The anti-apoptotic protein HAX-1 interacts with SERCA2 and regulates its protein levels to promote cell survival. *Mol biol cell*. 2009;20(1):306–318.

Warren JT, Cupo RR, Wattanasirakul P, et al. Heterozygous variants of CLPB are a cause of severe congenital neutropenia. *Blood.* 2022;139(5):779-791.

Wortmann SB, Ziętkiewicz S, Kousi M, et al. CLPB mutations cause 3-methylglutaconic aciduria, progressive brain atrophy, intellectual disability, congenital neutropenia, cataracts, movement disorder. *Am j hum genet.* 2015;*96*(2): 245-257

Wortmann SB, Ziętkiewicz S, Guerrero-Castillo S, et al. Neutropenia and intellectual disability are hallmarks of biallelic and de novo CLPB deficiency. *Genet med.* 2021;23(9):1705-1714.

Wai T, Saita S, Nolte H, Müller S, et al. The membrane scaffold SLP2 anchors a proteolytic hub in mitochondria containing PARL and the i‐AAA protease YME1L. *EMBO rep*, 2016;17(12):1844-1856.

Wei Y, Chiang WC, Sumpter R, Mishra P, Levine B. Prohibitin 2 is an inner mitochondrial membrane mitophagy receptor. *Cell*, 2017;*168*(1):224-238.

Yan J, Ma C, Cheng J, Li Z, Liu C. HAX-1 inhibits apoptosis in prostate cancer through the suppression of caspase-9 activation. *Oncol rep*, 2015;34(5):2776–2781

Yap SV, Koontz JM, Kontrogianni-Konstantopoulos A.. HAX-1: a family of apoptotic regulators in health and disease. *J cell phys.* 2011;226(11):2752–2761.
